# Peri-incisional perfusion does not differ between running versus interrupted Allgöwer-Donati suture technique in ankle fracture surgery: a pilot randomized controlled trial of wound perfusion

**DOI:** 10.1097/OI9.0000000000000097

**Published:** 2021-02-18

**Authors:** Sorawut Thamyongkit, Kitchai Luksameearunothai, Babar Shafiq, Erik A. Hasenboehler

**Affiliations:** a Department of Orthopaedic Surgery, The Johns Hopkins University, Baltimore, MD; b Chakri Naruebodindra Medical Institute, Faculty of Medicine Ramathibodi Hospital, Mahidol University, Bangkok, Thailand.

**Keywords:** ankle fracture, interrupted Allgöwer-Donati, running Allgöwer-Donati, skin perfusion, wound complications

## Abstract

**Objectives:**

To compare peri-incisional perfusion, perfusion impairment and wound closure time between the conventional interrupted Allgöwer-Donati (IAD) technique and a modified running Allgöwer-Donati (RAD) technique in ankle fracture surgery.

**Design:**

Prospective, randomized controlled clinical trial.

**Setting:**

Level I and II trauma centers.

**Patients:**

Twenty-five healthy adults with ankle fractures (AO/OTA 44-A, 44-B, or 44-C) between November 2017 and December 2018. (Of 26 patients enrolled in this study, 1 was lost to follow-up.)

**Intervention:**

Participants were randomized into the IAD or the RAD group (13 patients each). All participants were followed for at least 3 months after surgery to assess for wound complications.

**Main Outcome Measurements:**

Skin perfusion was assessed immediately after wound closure with laser-assisted indocyanine green angiography. Wound closure time, mean incision perfusion, and mean perfusion impairment were measured and compared with analysis of variance. Alpha = 0.05.

**Results:**

The RAD technique was significantly faster in terms of mean (± standard deviation) wound closure time (6.2 ± 1.4 minutes) compared with the IAD technique (7.3 ± 1.4 minutes) (*P* = 0.047). We found no differences in mean incision perfusion and mean perfusion impairment (all, *P* > 0.05).

**Conclusion:**

The IAD and RAD techniques resulted in similar peri-incisional perfusion and perfusion impairment. Closure time was significantly shorter for the RAD technique compared with the IAD technique.

**Level of Evidence:**

I

## 1 Introduction

For periarticular fractures, especially those about the ankle, open reduction and internal fixation is typically required to accomplish these goals. The soft-tissue envelope in this area is prone to postoperative wound complications because of the thin soft-tissue envelope. Cost of care increases substantially in cases of postoperative wound breakdown and infection. Among many factors within the surgeon's control, wound closure technique can substantially affect outcome.^[1,2]^

Several suture techniques for wound closure performed in a running or interrupted fashion have been described.^[3–11]^ The interrupted Allgöwer-Donati (IAD) suture technique (i.e., interrupted modified vertical mattress suture technique) has been recommended for at-risk wounds, flaps, areas of severe contusion, and regions with poor perfusion, such as around the ankle.^[12]^ The IAD technique minimizes disturbance to peri-incisional microcirculation by permitting a generous soft-tissue surface in each suture loop, resulting in lower risk of postoperative wound complications.^[10,13–16]^

To our knowledge, the degree of peri-incisional skin perfusion and the time it takes to close a wound using the modified running Allgöwer-Donati (RAD) technique have not been reported. Our purpose was to assess peri-incisional perfusion and perfusion impairment of surgical wounds, as well as closure time between the IAD and RAD suture techniques in ankle fracture surgery. We hypothesized that the RAD technique would be faster to perform and would result in similar cutaneous blood perfusion and wound complication rates compared with the IAD technique.

## 2 Materials and methods

### 2.1 Participant enrollment and randomization

This prospective, randomized controlled clinical trial was performed at our level I and II trauma centers and was approved by the Johns Hopkins Hospital Institutional Review Board (No.00127513). Informed consent was obtained from each participant by 1 of 2 board-certified orthopaedic trauma surgeons.

**Figure 1:**
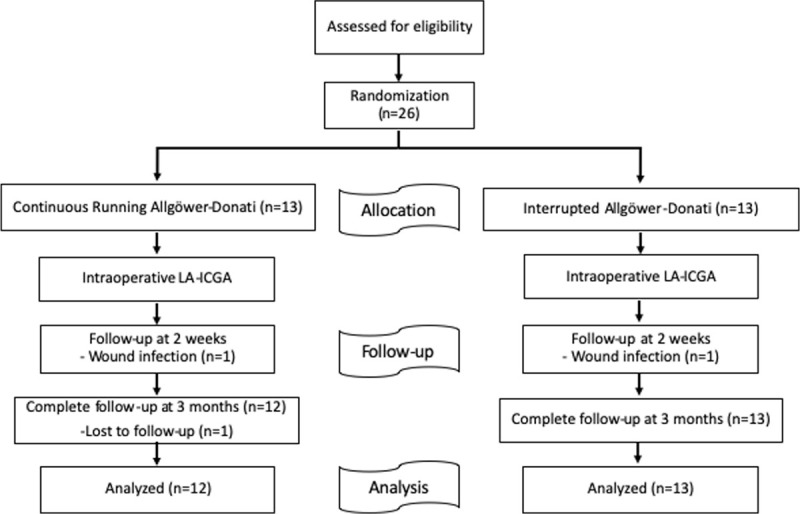
Study flow diagram of 26 patients presenting with an ankle fracture (AO/OTA 44-A, 44-B, or 44-C) between November 2017 and December 2018. LA-ICGA, laser assisted-indocyanine green angiography.

Patients who presented with an ankle fracture (AO/OTA 44-A, 44-B, or 44-C) between November 2017 and December 2018 were recruited to participate in the study (Fig. 1).^[17]^ We included patients aged 18 to 65 years whose planned treatment was open reduction and internal fixation. We excluded patients with a history of iodine allergy, steroid use, known peripheral vascular disease, diabetes mellitus, smoking, human immunodeficiency virus, hepatitis C or B, or syphilis, as well as those taking anticoagulants. We also excluded patients who did not provide written informed consent.

Permuted-block randomization was used to allocate participants to the IAD or RAD technique in a 1:1 ratio. In all cases, a lateral ankle incision was designated as the site for IAD or RAD suture application.

### 2.2 Participant characteristics

The 2 groups did not differ significantly in terms of age, sex, BMI value, time to surgery, incision length, or fracture classification (Table 1). This lack of difference between the 2 groups supports the effectiveness of treatment randomization.

**Table 1:** Characteristics of 25 participants with ankle fractures randomized by suture technique, November 2017 to December 2018

	All participants (n = 25)		RAD group (n = 12)		IAD group (n = 13)		
				
Characteristic	Mean (SD)	N (%)	Mean (SD)	N (%)	Mean (SD)	N (%)	*P*
Age, years	40 (15)		38 (14)		42 (15)		0.52
Female sex		16 (64)		8 (67)		8 (62)	0.79
BMI, kg/m^2^	31 (7.7)		33 (9.4)		29 (5.5)		0.26
Time to surgery, days	13 (7.1)		16 (7.1)		12 (6.6)		0.12
AO/OTA classification							
44-A		1 (4.0)		1 (8.3)		0 (0)	0.28
44-B		18 (72)		7 (58)		11 (85)	
44-C		6 (24)		4 (33)		2 (15)	
Incision length, cm	11.5 (2.3)		12.4 (2.4)		10.7 (2.0)		0.67
Follow-up time, months	3.4 ± 2.7		3.0 ± 2.3		3.8 ± 2.7		0.48

BMI = body mass index; SD = standard deviation.

### 2.3 Operative procedures

The surgery was delayed until swelling subsided and good wrinkling signs were present. Surgery was performed with the patient in the supine or lateral position on a radiolucent table. The anesthesiologist chose the anesthetic method. The operative lower extremity was prepared for surgery and draped, and the skin was marked for a lateral approach. A tourniquet was used throughout the procedure. A lateral incision was made, and skin bridges of at least 7 cm long were used when multiple incisions were needed. The 7-cm skin bridge has been recommended since publication of the second edition of the AO manual.^[18]^ In a prospective study, Howard et al^[19]^ found that a skin bridge of less than 7 cm may be safe for wound healing in tibial plafond surgery. On the basis of this evidence and to standardize our study protocol, we ensured that a 7-cm or longer skin bridge was used. Fracture reduction and fixation was performed per the AO technique, using 1 or more lag screws and a neutralization plate. Medial and posterior malleolus fractures, if present, were stabilized with a buttress plate and/or lag screws. After fracture fixation, an external rotation or Cotton stress test was performed, and the syndesmosis was reduced and stabilized as indicated.

**Figure 2:**
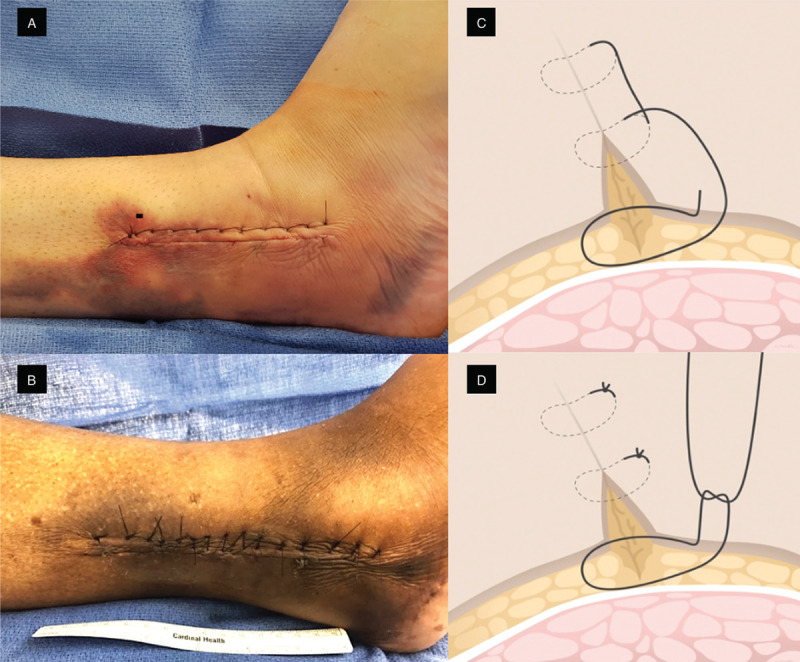
Postoperative photographs of running Allgöwer-Donati suture technique (A) and interrupted Allgöwer-Donati suture technique (B). Illustrations of running Allgöwer-Donati suture technique (C) and interrupted Allgöwer-Donati suture technique (D).

Wounds were then closed in layers, using number 2-0 resorbable suture (Vicryl, Ethicon Inc, Somerville, NJ). The skin was closed using the IAD or RAD technique, per randomization assignment. In all cases, number 3-0 nylon suture (Ethicon Inc, Somerville, NJ) was used for skin closure. For the IAD group, the sutures were placed at 1-cm intervals. For the RAD group, the suture was done in a similar fashion but the knot was not tied until the end of incision. The suture was looped under each previous stitch and placed at 1-cm intervals (Fig. 2). The surgery and wound closure were performed by 1 of 2 board-certified orthopaedic trauma surgeons (BS and EAH).

### 2.4 Perfusion testing

**Figure 3:**
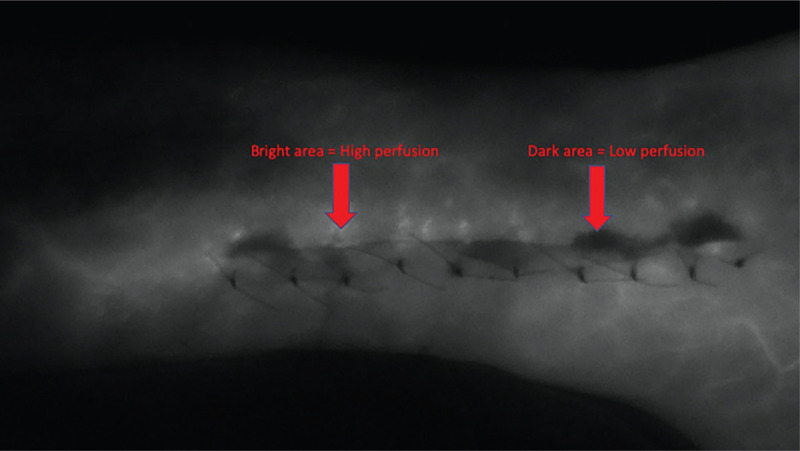
Intraoperative image along the lateral ankle incision using the SPY Elite fluorescent imaging system (NOVADAQ Technologies Inc, Bonita Springs, FL). Bright areas indicate high perfusion. Dark areas indicate low perfusion.

After wound closure and tourniquet release, participants underwent intraoperative peri-incisional perfusion measurement. Measurement was done by laser-assisted indocyanine green angiography (LA-ICGA) using the SPY Elite fluorescent imaging system (NOVADAQ Technologies Inc., Bonita Springs, FL). The LA-ICGA system is widely used by plastic surgeons to quantify perfusion of soft tissue and determine skin and flap viability. The perfusion measurements were made according to manufacturer's recommended technique and similar to previous studies.^[14,15,20]^ The LA-ICGA camera was positioned directly facing the ankle incision at a distance indicated by overlapping laser lights mounted to the camera for this purpose. The anesthesiologist then injected a 5-mL bolus of indocyanine green dye followed by a 10-mL saline flush. Finally, with the overhead operating room lights turned off and a fluorescent light projected onto the incision, perfusion was recorded by video for 2 minutes. The darker areas in the resulting video represent lower perfusion and brighter areas represent higher perfusion (Fig. 3).

**Figure 4:**
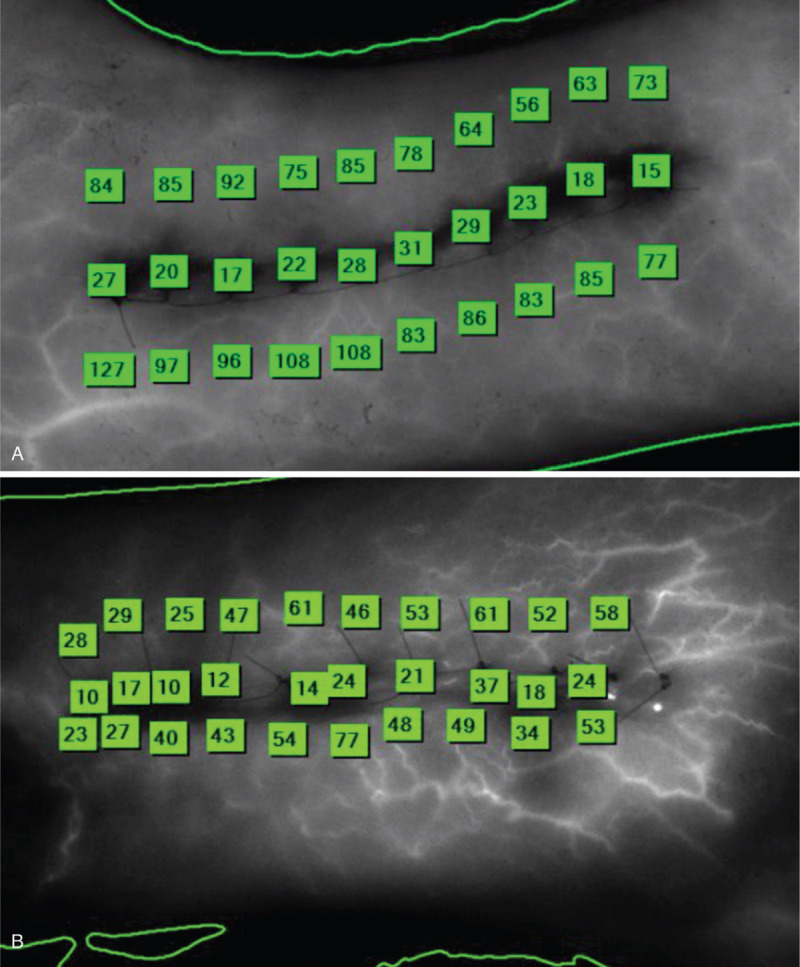
Intraoperative images with fluorescence units representing peri-incision perfusion measured using the SPY Elite fluorescence imaging system (NOVADAQ Technologies Inc, Bonita Springs, FL). (A) Running Allgöwer-Donati suture technique. (B) Interrupted Allgöwer-Donati suture technique.

According to Shannon et al,^[14]^ peak perfusion (maximum fluorescence during the arterial phase) occurs during the first 20 seconds, and baseline perfusion (the plateau phase of fluorescence, in which the arterial and venous phases of perfusion are in equilibrium) occurs at 70 seconds (Fig. 4).^[21]^ Therefore, we quantified perfusion at 20 seconds and 70 seconds into recording by using the LA-ICGA scale,^[20]^ which ranges from 0 to 255 fluorescence units. Perfusion was measured at 30 data points for each incision: 10 points immediately adjacent to the incision, 10 points 1 cm anterior to the incision, and 10 points 1 cm posterior to the incision.

### 2.5 Postoperative care

After perfusion testing, a well-padded splint was applied. Standard postoperative care was applied in both groups. We used the postoperative protocol of the senior authors (BS and EAH) to standardize postoperative care. At 2 weeks postoperatively, patients underwent a wound check and suture removal, as well as initiation of range-of-motion exercises. Progressive weightbearing began at approximately 6 weeks to minimize soft tissue complications.

### 2.6 Outcome measures

#### 2.6.1 Closure time

Closure time was measured from the start of skin suturing until the last suture knot was tied.

#### 2.6.2 Mean incision perfusion (MIP)

To minimize confounding, we assessed only the cutaneous blood perfusion of the lateral skin incision over the fibula for both perfusion measures. MIP was defined by Wyles et al^[21]^ as the mean fluorescence of 10 points adjacent to the incision. MIP is used to quantify absolute perfusion to the incision, with higher values indicating greater blood flow. We compared MIP between the IAD and RAD groups.

#### 2.6.3 Mean perfusion impairment (MPI)

MPI is calculated as the difference between the mean of each pair of points anterior and posterior to the incision and the points adjacent to the incision.^[15]^ Smaller MPI values indicate less perfusion impairment. We compared MPI between the IAD and RAD groups.

#### 2.6.4 Complications

Wound dehiscence, wound infection, and other complications were recorded for at least 3 months after surgery by surgeons and physician assistants. The mean duration of follow-up was 3.8 ± 2.7 months in the IAD group and 3.0 ± 2.3 months in the RAD group (*P* = 0.48).

### 2.7 Statistical methods

According to Shannon et al,^[14]^ a sample size of 10 in each group has 80% power to detect a difference of 15 fluorescence units for MPI and MIP, with a 2-sided alpha value of 0.05. Normally distributed continuous data (i.e., age, BMI value, time to surgery, MPI, and MIP) are reported as means ± standard deviations and compared using Student *t* tests. Categorical data (i.e., patient sex, AO/OTA classification, number of wound complications, and revision surgery) were compared using Pearson chi-squared tests. Primary outcomes (MPI and MIP at baseline perfusion) were analyzed separately. The same analytic strategy was used for the secondary outcomes (MPI and MIP at peak perfusion). The analysis was conducted using Student *t* tests with MIP or MPI as the dependent variable and closure techniques as the independent variable. *P* values < 0.05 were considered significant. Analyses were conducted using SPSS, version 18.0, statistical software (PASW Statistics for Windows, SPSS Inc, Chicago, IL).

## 3 Results

A total of 26 healthy adults with ankle fractures were enrolled in this study. One patient who could not complete postoperative follow-up of at least 3 months was excluded.

### 3.1 Closure time

The mean (± standard deviation) closure time for the RAD group (6.2 ± 1.4 minutes) was significantly shorter than for the IAD group (7.3 ± 1.4 minutes) (*P* = 0.047).

### 3.2 Perfusion

MIP and MPI did not differ significantly between groups. Baseline MIP was 19 ± 11 fluorescence units in the RAD group and 24 ± 16 fluorescence units in the IAD group (*P* = 0.32). Baseline MPI was 25 ± 16 fluorescence units in the RAD group and 24 ± 20 fluorescence units in the IAD group (*P* = 0.93). Peak MIP was 15 ± 9.9 and 17 ± 12 fluorescence units in the RAD and IAD groups, respectively (*P* = 0.75). Peak MPI was 51 ± 39 and 51 ± 41 fluorescence units in the RAD and IAD groups, respectively (*P* = 0.996; Table 2).

**Table 2:** Closure time, baseline and peak perfusion, and complications by RAD (n = 12) or IAD (n = 13) wound closure technique

	Mean ± SD			Mean ± SE			N		
			
Parameter	RAD group	IAD group	*P*	RAD group	IAD group	*P*	RAD group	IAD group	*P*
Closure time, min.	6.2 ± 1.4	7.3 ± 1.4	0.047	5.8 ± 0.31^∗^	7.7 ± 0.30^∗^	<0.01	NA	NA	NA
Baseline perfusion^†^									
Incision perfusion	19 ± 11	24 ± 16	0.32	19 ± 4.2^‡^	25 ± 4.1^‡^	0.31	NA	NA	NA
Perfusion impairment	25 ± 16	24 ± 20	0.93	23 ± 5.0^‡^	26 ± 4.8^‡^	0.18	NA	NA	NA
Peak perfusion^†^									
Incision perfusion	15 ± 9.9	17 ± 12	0.75	14 ± 3.2^‡^	18 ± 3.1^‡^	0.41	NA	NA	NA
Perfusion impairment	51 ± 39	51 ± 41	0.996	46 ± 12^‡^	55 ± 12^‡^	0.60	NA	NA	NA
Complications									
Wound complications	NA	NA	NA	NA	NA	NA	0	1	0.37
Revision surgeries	NA	NA	NA	NA	NA	NA	1	0	0.29

NA = not applicable; SD = standard deviation; SE = standard error.

∗ Adjusted for age, sex, body mass index value, and incision length.

† Expressed in fluorescence units, which range from 0 to 255.^[20]^

‡ Adjusted for age, sex, body mass index value, and time to surgery.

### 3.3 Complications

One participant in the IAD group developed a superficial infection of the lateral incision and was treated successfully with oral antibiotics. One participant in the RAD group underwent revision surgery because of syndesmotic fixation failure. No wound complications occurred in the RAD group. The groups did not differ significantly by rate of wound complications (*P* = 0.37) or revision surgery (*P* = 0.29).

## 4 Discussion

In this study of 2 wound closure techniques in ankle fracture surgery, we found no significant differences in peri-incisional perfusion between RAD and IAD suture techniques. In addition, the RAD technique was faster than the IAD technique and resulted in similar peri-incisional perfusion and similar complication rates. The RAD technique allows wound closure with minimal soft-tissue handling compared with the IAD technique, in which the epidermal layer must be everted more aggressively because of the tightly knotted previous adjacent stitch. Although the RAD technique took significantly less closure time compared with the IAD technique, this difference may not be clinically relevant.

Wound complications are associated with worse functional outcomes, longer time to rehabilitation, and greater treatment costs. The reported rates of wound dehiscence and wound infection after ankle fracture repair are 10% and 1.8% to 8.6%, respectively.^[22–27]^ Higher rates can be expected in patients with diabetes. Patient factors, such as diabetes, peripheral vascular disease, initial trauma, and nicotine use, may increase the risk of wound complications.^[28]^ Factors within the surgeon's control that may reduce these complications include careful soft-tissue handling and wound closure. The ideal wound closure technique should be easy and quick and should facilitate perfusion to support wound healing. Several studies have compared sutured techniques and suture materials.^[4,7,13–16]^

The running suture technique is widely used in current practice. This technique has advantages compared with the interrupted suture technique, in that it is easier and faster to perform, and suture tension can be adjusted evenly along the incision.^[29,30]^ However, there is concern that running suture technique may restrict peri-incisional perfusion. Wound perfusion has been recognized as a major factor affecting wound healing. The deposition and cross-linking of collagen are complex processes during wound healing, and these processes are influenced by oxygen tension and blood perfusion.^[31]^ Because of this concern, the IAD suture technique has been recommended by the AO-Association of the Study of Internal Fixation Group for closure of skin flaps and in compromised skin conditions.^[12]^ In previous studies, the IAD technique resulted in less perfusion impairment compared with traditional vertical mattress sutures, horizontal mattress sutures, and surgical staples.^[10,14,21]^ Thus, we decided to compare the IAD and the new RAD techniques in this study.

Limitations of this study include a small sample size, which may have prevented identification of differences in wound complication rates between groups. Second, tissue perfusion was measured immediately after wound closure with no additional follow-up measurement. Peri-incisional perfusion may change as swelling develops, but this was not assessed. Third, we were unable to blind the surgeons, participants, and wound evaluators (surgeons and physician assistants) to suture techniques. A major strength of our study is that it was a randomized trial, which minimized bias. We used participant selection criteria, excluding patients with factors known to affect wound healing, including diabetes mellitus, tobacco use, respiratory disorders, obesity, collagen disorders, malnutrition, malignancy, and drug use (steroid and aspirin). However, these patients do not represent typical practice, in which patients with underlying conditions would be included. A larger, prospective randomized study is needed to determine differences and compare complications between the 2 suture techniques.

## 5 Conclusion

In ankle fracture surgery, the RAD suture technique is faster to perform and results in similar peri-incisional wound perfusion compared with the IAD suture technique. We found no difference in wound complication rates between the 2 suture techniques at 3-month follow-up. The RAD technique may be a faster closure option with no increased wound risk for incisions about the ankle.
